# A Novel Theory-Based Virtual Reality Training to Improve Patient Safety Culture in the Department of Surgery of a Large Academic Medical Center: Protocol for a Mixed Methods Study

**DOI:** 10.2196/40445

**Published:** 2022-08-24

**Authors:** Lukasz M Mazur, Amro Khasawneh, Christi Fenison, Shawna Buchanan, Ian M Kratzke, Karthik Adapa, Selena J An, Logan Butler, Ashlyn Zebrowski, Praneeth Chakravarthula, Jin H Ra

**Affiliations:** 1 Division of Healthcare Engineering, Department of Radiation Oncology School of Medicine University of North Carolina Chapel Hill, NC United States; 2 Industrial Engineering Department School of Engineering Mercer University Macon, GA United States; 3 Academic Technology Services School of Medicine University of North Carolina Chapel Hill, NC United States; 4 Department of Surgery School of Medicine University of North Carolina Chapel Hill, NC United States; 5 Department of Computer Science University of North Carolina Chapel Hill, NC United States

**Keywords:** virtual reality training, patient safety culture, patient safety events, sensemaking, high reliability organizations

## Abstract

**Background:**

Preventable surgical errors of varying degrees of physical, emotional, and financial harm account for a significant number of adverse events. These errors are frequently tied to systemic problems within a health care system, including the absence of necessary policies/procedures, obstructive cultural hierarchy, and communication breakdown between staff. We developed an innovative, theory-based virtual reality (VR) training to promote understanding and sensemaking toward the holistic view of the culture of patient safety and high reliability.

**Objective:**

We aim to assess the effect of VR training on health care workers’ (HCWs’) understanding of contributing factors to patient safety events, sensemaking of patient safety culture, and high reliability organization principles in the laboratory environment. Further, we aim to assess the effect of VR training on patient safety culture, TeamSTEPPS behavior scores, and reporting of patient safety events in the surgery department of an academic medical center in the clinical environment.

**Methods:**

This mixed methods study uses a pre-VR versus post-VR training study design involving attending faculty, residents, nurses, technicians of the department of surgery, and frontline HCWs in the operation rooms at an academic medical center. HCWs’ understanding of contributing factors to patient safety events will be assessed using a scale based on the Human Factors Analysis and Classification System. We will use the data frame theory framework, supported by a semistructured interview guide to capture the sensemaking process of patient safety culture and principles of high reliability organizations. Changes in the culture of patient safety will be quantified using the Agency for Healthcare Research and Quality surveys on patient safety culture. TeamSTEPPS behavior scores based on observation will be measured using the Teamwork Evaluation of Non-Technical Skills tool. Patient safety events reported in the voluntary institutional reporting system will be compared before the training versus those after the training. We will compare the Agency for Healthcare Research and Quality patient safety culture scores and patient safety events reporting before the training versus those after the training by using descriptive statistics and a within-subject 2-tailed, 2-sample *t* test with the significance level set at .05.

**Results:**

Ethics approval was obtained in May 2021 from the institutional review board of the University of North Carolina at Chapel Hill (22-1150). The enrollment of participants for this study will start in fall 2022 and is expected to be completed by early spring 2023. The data analysis is expected to be completed by July 2023.

**Conclusions:**

Our findings will help assess the effectiveness of VR training in improving HCWs’ understanding of contributing factors of patient safety events, sensemaking of patient safety culture, and principles and behaviors of high reliability organizations. These findings will contribute to developing VR training to improve patient safety culture in other specialties.

## Introduction

### Background

High-quality health care requires continual efforts to decrease the incidence of medical errors [1]. Surgical patients are at particular risk for error-associated adverse outcomes, given the invasiveness of undergoing surgery [2]. It is estimated that annually over 4000 harmful surgical errors are preventable nationally [1]. Surgical errors may lead to temporary or permanent harm to the patient (eg, physical, emotional, or even death) and may cause harm to care providers (eg, second victim) [3]. For example, unintended retention of foreign objects, which is estimated to occur at least once in every 5500 surgeries, [3] may lead to reoperation, increased hospital length of stay, and sepsis [3]. Moreover, the average additional cost related to unintended retention of foreign objects is estimated to be more than US $200,000 per incident [4]. The common root causes of surgical errors reported to the Joint Commission include the absence of policies and procedures, problems with hierarchy and intimidation, failure to communicate with physicians, and failure of staff to communicate relevant patient information [5]. Additionally, factors such as high workload, time pressure, and the resulting burnout are associated with higher rates of errors. These root causes have been difficult to address, as complex care delivery systems tend to “drift” over time creating new sources of failures and failure pathways [5].

Safety problems in health care persist because they are multifactorial and complex. A barrier to improving safety appears to be a broad lack of appreciation for the underlying causes of safety problems and everyone’s role in contributing to these problems. There is a need to collectively strive to build a “culture of patient safety,” with excellence at all levels, as is common in high reliability organizations (HROs) [6]. An evolving concept in the area of quality and safety is sensemaking, which is an action taken in response to an individual’s interpretation of ambiguous events. Sensemaking is essential to achieve commitment to HRO-like thinking and behaviors [7]. Sensemaking has been shown to improve patient safety by improving health care workers’ (HCWs’) commitment to a culture of patient safety and the reliability of an organization [7]. For example, by improving sensemaking, bedside nurses can evaluate and determine the appropriate response to safety concerns expressed by patients or their families [8].

Current interventions to improve safety largely address the sharp end of the error. For example, at the resident level, the Accreditation Council for Graduate Medical Education requires residents, including surgical residents, to participate in interprofessional patient safety training and activities such as performing root cause analyses and reporting patient safety events, with evidence that these requirements lead to positive patient safety improvements [9]. Moreover, simulation-based training for attending surgeons appeared to decrease subsequent malpractice claims [10]. Most of these efforts, however, focus on skills needed at the sharp end of the error (eg, communication and teamwork in the operating room, technical skills) yet lack the training on the faulty systems (eg, Swiss cheese model [SCM] [11] of error prevention), the culture of patient safety, and the unreliable thinking and behaviors that lead people to make mistakes or fail to prevent them [12].

The use of virtual reality (VR) training in health care is becoming an increasingly feasible and effective method for training. Research suggests that VR training in health care can improve technical and nontechnical skills (eg, team communication, teamwork), planning and performing surgery, and medical diagnosis [13]. Most importantly, VR appears to be particularly well suited to be modified to address education on nontechnical skills, including the holistic view of the culture of patient safety and high reliability, as well as the sensemaking of patient safety events. Although such VR-based training has not been widely applied in health care, it has been successfully applied in construction [14]. Alternative approaches to providing this type of training within health care are perhaps suboptimal. Major health care systems currently rely on a webpage-based learning management system and the associated PowerPoint presentations. While useful, VR may provide this information more interactively and engagingly. Thus, there is a need to develop a patient safety training program that promotes understanding and sensemaking toward the holistic view of the culture of patient safety and high reliability. Furthermore, the training program must help HCWs better understand the overall extent of patient safety from the blunt to the sharp end of the error and, consequently, emphasize a culture of improvement at all levels of the organization.

VR can give HCWs immersive first-person access to realistic health care scenarios where adverse events occur, thereby enabling a deeper experiential learning exposure to the contextual realities surrounding such events, without posing any risk or harm to a real patient. As a strategic training solution, VR may enable the provision of complex health care training scenarios that would otherwise be inaccessible as real-world observations owing to their unpredictable and high-risk nature, too resource-intensive to re-enact in a live clinical training setting on a per learner basis, and ineffective as a passive 2D module, where the goal of the training is to experientially connect learners to the lived, contextualized realities surrounding an adverse event for situational sensemaking. Among these difficult to train for adverse events is a surgical error in action. Errors are by nature unintended and thus cannot be anticipated during clinical shadowing. Specifically, surgical errors occur in an operating room, where strict policies for patient safety make observation impossible for most learners and anyone outside of the surgical team itself. This reveals a key experiential exposure obstacle: access to the real context in which a surgical error occurs. Although live mock simulations using standardized patients are used for certain health care scenarios, the resources involved in realistically recreating a surgical scenario include the time and coordination for full representation of a surgical team, time in an operating room, or the creation of a realistic representation of one, patient representation, and an array of special effects that must coincide with the error and necessitate cleanup/reset for each run-through. These resource requirements are immense and thus neither cost-effective nor scalable for multiple realistic per-learner exposures. Additionally, such a live mock simulation continues to fall short in meeting the holistic need, providing insight into the larger contributing factors and consequences that exist beyond the operating room itself. Meanwhile, traditional 2D content may be used in the topical illustration of a surgical error, but by nature, exists as a passively consumed resource disconnected from learners’ spatially contextualized, experiential reality. Although this disconnection may or may not impact certain forms of learning such as the ability to recall facts, higher-level central processing of theoretical events, including a simulated engagement in sensemaking of complex situations, may benefit from more experiential learning approaches. Experiential learning involves a personalized and cognitive engagement with the learning content, emphasizing the linkage between learning and its “real world” context. If HCWs are to engage in sensemaking activities regarding the complex lived realities that are connected to adverse events, VR may offer advantages to 2D content in that it allows HCWs to access an immersed perspective of the event as it would occur before one’s own eyes.

We have developed an innovative VR-based training to improve patient safety culture and HCWs’ understanding of factors contributing to patient safety events, sensemaking, and HRO-like thinking and behaviors. This training is rooted in Reason’s SCM [11], which demonstrates how system failures are often the result of a combination of factors. The training is designed for HCWs to build deep emotional connections and to facilitate understanding and sensemaking toward the culture of patient safety and HRO-like thinking and behaviors [12,15]. Our goal is to implement and evaluate this innovative VR training at a large academic medical system in the southeast United States. To the best of our knowledge, this VR training is the first-of-its-kind high-fidelity model focused on the culture of patient safety and HRO thinking and behaviors and designed to help HCWs make sense of patient safety events in health care.

The VR training tool utilizes multiple 360° scenes arranged in a narrative format and delivered within an interactive VR-input enabled application. The 360° videos were recorded with 2 different 360° cameras: first-person perspectives were recorded with a head-mounted GoPro Max in 5.6K resolution and a third-person observer view was recorded for each scene with the Insta360 Titan in 11K resolution. The GoPro Max was selected for the first-person perspectives owing to its small size for head mounting and stabilization features. The Insta360 Titan was also used for its ability to capture the full scene in a higher resolution/level of detail. Each scene within the simulation features a room view as well as a first-person perspective view. The 360° videos include scenes within the operating room where the adverse event occurs, different locations within the hospital, different locations within home environments, and a board room where hospital policies are discussed; each of these locations serves to explore the complex realities of the various perspectives connected to the adverse event. The 360° videos were edited using Adobe Premiere Pro and Boris FX for both cinematic scene composition and special effects as needed, and each scene was exported as its own video file for interactive scene selection options in-app. The initial development of the interactive VR app used 3DVista—a visual scripting tool for the creation of interactive 360° video applications. The 3DVista software was selected for a quicker design iteration process, publishing a product that can be delivered via both WebXR on any device and as a sideloaded local app on the standalone VR headset itself. The app features a welcome user interface menu providing an overview of the simulation content and purpose to provide a background context as well as instructions for VR use prior to starting the simulation. Buttons are present within the app to allow for perspective switching within each scene, as learners can explore the perspective-based contributions and effects surrounding the surgical error featured in the simulation. Within each scene, a few interactive hotspots are present to allow users to further observe the contextual details present. After testing our initial VR tool design, the app’s final design choice will be rebuilt in the Unity game engine to enable future C#-based customizations as needed, such as enabling potential eye-tracking-dependent research questions and potential learning management system integrations. This tool is being developed in-house by our research team on behalf of the University of North Carolina (UNC) School of Medicine IT Instructional Media Services.

### Objectives

The overall aim of this study is to create a VR-simulated environment where HCWs profoundly experience and learn the concepts of patient safety culture and principles and behaviors of HROs. This will be created in a human factors laboratory and will be used to train HCWs in a large academic medical center. The specific aims of this study are as follows: (1) to assess the effect of innovative VR training on HCWs’ understanding of contributing factors to patient safety events, sensemaking of patient safety culture, and principles and behaviors of HROs in the laboratory environment and (2) to assess the effect of innovative VR training on patient safety culture, TeamSTEPPS behavior scores, and reporting of patient safety events in the surgery department of an academic medical center in clinical settings.

We hypothesize that HCWs’ understanding of contributing factors to patients’ safety events (*hypothesis 1a*), sensemaking process of patient safety culture, and principles of HROs (*hypothesis 1b)* will improve from pre-VR to post-VR training. Further, we hypothesize that there will be an increase in patient safety culture score (*hypothesis 2a)*, TeamSTEPPS behavior scores (*hypothesis 2b),* and reporting of patient safety events (*hypothesis 2c)* in pre-VR versus post-VR training.

## Methods

### Study Design

This study uses a mixed methods approach and a pre-VR versus post-VR training study design. In the first phase of the study, participants will first read a description of a patient safety event and then be asked to evaluate the contributing factors associated with the event. Next, participants will experience a patient safety event in an immersive interactive VR environment, followed by evaluating their understanding of the contributing factors associated with the event. We will use the data frame theory framework [16] supported by a semistructured interview guide to capture the sensemaking process of patient safety culture and principles of HROs for an HCW. We will conduct interviews with the participants after they have experienced the VR training program. The second phase of the study involves comparing changes in the culture of patient safety, TeamSTEPPS behavior scores, and reporting of patient safety events in pre-VR versus that in post-VR training.

### Participants

The participants for this study are attending faculty, residents, nurses, and technicians working in the Department of Surgery at UNC Health. This department includes 68 faculty and 75 residents. Residents are divided into general surgery, cardiothoracic, vascular, and plastic surgery. Within general surgery are additional faculty divisions (pediatric, acute care, transplant, oncology, gastrointestinal, burns). Faculty practice mostly at UNC’s main medical center, but we also have some faculty at Hillsborough and Rex. All frontline HCWs from operation rooms and the UNC Department of Surgery will also be invited to participate in the study. We will exclude traveler nurses, temporary employees, and administrative staff from this study. An onsite research team member will answer questions from prospective participants, and the principal investigators’ contact information will be available on the flyer. A previously used scripted protocol and emails sent through a departmental listserv will be used to inform prospective participants about the study. If they choose to participate, we will obtain consent from the participant before the first phase of the study. An incentive of US $50 will be provided to the participants for the first phase of the study.

### Setting

The first phase of the study, which provides participants with experience of a patient safety event in an immersive interactive VR environment, will be conducted in the Human Factors Laboratory located in the Department of Radiation Oncology at UNC.

### Intervention

The VR training is guided by Reason’s SCM [11,15] and Human Factors Analysis and Classification System (HFACS) [17,18] and targets each layer in the HFACS model. We utilized state-of-the-art filming equipment to capture a 360° view of the event and the perspective of those involved in the event. We built the scripts for the scenes, recruited actors (attendings, residents, students, administrators) with lived experiences in health care to help with the filming, identified filming locations, rehearsed all the scenes, and filmed our scenes. We expect to assemble the complete training program and have the final product ready by July 2022. This training will be delivered to the participants using a VR head-mounted display to ensure an immersive environment. Specifically, we will use Pico Neo 3 Pro Eye [19] with 6DoF VR hardware/software to implement the VR training.

### Measures

#### Primary Measures

##### Understanding of Contributing Factors

Participants’ understanding of contributing factors to an event is assessed using a scale based on the HFACS (Multimedia Appendix 1).

##### Sensemaking of Patient Safety Culture and Principles of HROs

Participants’ understanding of patient safety culture and principles of HROs are assessed by conducting 2 semistructured interviews with each participant after reading the patient safety event description and after completing the VR training. The purpose is to explore participants’ baseline understanding of patient safety culture and principles of HROs before taking the training and to examine their experience with the training, including how did the training change their understanding of patient safety culture and principles of HROs. Interviews are guided by a phenomenological approach, which follows the paradigm of subjectivity and emphasizes the importance of understanding personal experience to gain insights into the sensemaking process of patient safety events concept as the individuals take the training program [16]. This approach has been applied in previous studies to examine the sensemaking process of making health care decisions [20]. Interview questions are presented in Multimedia Appendix 2. Face-to-face interviews for 10 minutes are conducted with each participant. If participants permit, interviews are audio-recorded and transcribed for analysis or else, the research team will take detailed handwritten notes.

#### Secondary Measures

##### Patient Safety Culture

Patient safety culture is assessed using the Agency for Healthcare Research and Quality (AHRQ) Survey on Patient Safety Culture (SOPS). AHRQ SOPS is a reliable and validated instrument to assess and capture all staff members’ perceptions on key components of patient safety culture, such as teamwork, staffing, work pace, organizational learning, response to error, clinical leader support for patient safety, communication about the error, communication openness, reporting patient safety events, hospital management support for patient safety, handoffs, and information exchange [21,22]. The Department of Surgery will take this survey after the intervention to allow for the pre-VR versus post-VR training comparison on patient safety culture.

##### TeamSTEPPS Behavioral Score

Teamwork is one of the key initiatives within patient safety that can transform the culture within health care. Studies suggest that communication and other teamwork skills are essential for the delivery of quality health care and for preventing and mitigating medical errors and patient injury and harm [23,24]. TeamSTEPPS behavioral scores are currently collected by the operation room nurses as part of regular operations by using the Teamwork Evaluation of Non-Technical Skills (TENTS) tool [25]. TENTS is a valid and reliable instrument to assess a variety of clinical teamwork events. It is a 13-item observational assessment tool used in clinical settings. These scores will be used to compare the results of pre-VR versus those of post-VR training [25].

##### Reporting of Patient Safety Events

Voluntary reporting of patient safety events is important for achieving the broad goal of error reduction. We will use the events reported in our institutional voluntary reporting system to compare the reporting of patient safety events before versus after the VR training. The events will be classified based on the AHRQ Common Format Harm score. Using the score (1 through 9) and the nature of harm, we will classify each patient safety event as serious safety events, precursor safety events, near-miss safety events, and unsafe condition safety events and compare before and after the VR training. Table 1 provides an overview of the measurement tools.

**Table 1:** Overview of the measurement tools.

Variable		Measure
**Primary outcomes**		
	Understanding of contributing factors	Human Factors Analysis and Classification System scale
	Sensemaking of patient safety culture and principles of high reliability organizations	Semistructured interview
**Secondary outcomes**		
	Patient safety culture	Agency for Healthcare Research and Quality Survey on Patient Safety Culture
	TeamSTEPPS behavioral scores	Teamwork Evaluation of Non-Technical Skills tool
	Reporting of patient safety events	Institutional voluntary reporting system
**Other variables**		
	Demographics	Questions

### Data Collection

All participants will first read a written description of the recorded patient safety event (see Multimedia Appendix 3). Then, they will take the VR training program.

*Hypothesis 1a:* All participants will rate the perceived contributing factors to the event using a scale based on the HFACS [17].

*Hypothesis 1b:* We will conduct 2 semistructured interviews with each participant: after reading the patient safety event description and after completing the VR training. The interviews are conducted to (1) explore their baseline understanding of patient safety culture and principles of HROs before taking the training and (2) to examine their experience with the training, including how did the training change their understanding of patient safety culture and principles of HROs.

*Hypothesis 2a:* The UNC Department of Surgery was scheduled to take the AHRQ SOPS survey in February and March 2022, which will serve as the baseline data. The UNC Department of Surgery agreed to take this survey again in February-March of 2023 (postintervention) to allow us for the pre-VR versus post-VR training comparison.

*Hypothesis 2b:* TeamSTEPPS behavioral scores are currently informally being collected by the operation room nurses as part of regular operations by using the TENTS tool. As needed, we will modify this data collection to fit the need of this proposal and support this data collection to ensure proper evaluation. We will use these scores to compare the results before versus after the VR training.

*Hypothesis 2c:* We will extract data on reporting of patient safety events from the Risk Level-6 solution platform (Safety Awareness For Everybody reporting) managed by the risk management department. We will access this data set and compare results before versus after the VR training.

### Data Analysis

*Hypothesis 1a*: A previous study showed that VR in a simulated learning environment can improve empathic clinical communication score by 0.3-1.15 points with a standard deviation (σ) of 0.3-1.1 [26]. To detect a statistically significant change in our study, a sample size of 68 participants is needed to obtain a medium-to-large effect size (*d*=0.5-0.8) with a power level of .80 and an alpha of .05 [26,27]. We will compare the number of identified contributing factors (after reading the event vs after VR training) by using a within-subject 2-tailed, 2-sample *t* test with the significance level set to .05.

*Hypothesis 1b*: We will recruit a subset of the participants who took the training to participate in an additional interview study. Based on the theoretical saturation concept and our prior experience, we estimate to conduct interviews with 20-28 participants (eg, 5-7 faculty, 5-7 nurses, 5-7 staff, 5-7 residents). However, we will continue to recruit additional participants until our data reach saturation [28]. Interview data will be analyzed using the phenomenological analysis method [29] to understand the different perspectives regarding patient safety culture and principles of HROs before taking the VR training and how the participants make sense of the training and the patient safety events they experienced during the training to adjust their understanding, behavior, and commitment to patients’ safety. The qualitative analysis will include 3 steps: (1) develop textual and structural descriptions for each interview, (2) composite all textual and structural descriptions, respectively, and (3) synthesize textual and structural meanings using the data frame theory of sensemaking [16].

*Hypothesis 2a*: Based on the AHRQ SOPS data collected by the Office of Quality Excellence at UNC Health in 2017 and 2019, we expect that we will need 75 participants to take the survey to obtain a medium effect size (*d*=0.5) with a power level of .80 and an alpha of .05 [27]. We will use 2-tailed, 2-sample *t* test to compare pooled and construct-based AHRQ SOPS scores before versus after the VR training.

*Hypothesis 2b:* We will use a 2-tailed, 2-sample *t* test to compare pooled and construct-based to assess changes in TENTS scores before versus after the VR training.

*Hypothesis 2c:* We will use a 2-tailed, 2-sample *t* test to compare reporting of patient safety events before versus after the VR training.

### Ethics Approval and Confidentiality of Data

This study received approval from UNC’s institutional review board in May 2022 (22-1150). Handling and storage of data will be done per the general data protection regulation and the institutional review board policies of UNC. Collected research data within this study includes questionnaires and interviews, collected by the researchers from the Department of Radiation Oncology and Surgery at UNC. All data will be deidentified by giving every participant a unique participant ID. All data from the questionnaires shall be stored in a protected folder that can be accessed only by the research team. Physical documents, for example, signed informed consent forms will be stored safely in the Human Factors Laboratory of the Department of Radiation Oncology, UNC. The recordings of the interviews will be stored in a secure encrypted folder that is accessible only to the research team. Research data and analyses will be stored after finishing the research project in accordance with the policies of UNC.

## Results

Participant recruitment will start in July-September 2022. Data collection for this study is expected to be completed by November 2023. The analysis will be conducted after data collection and is expected to be completed by December 2023. The results will be published in peer-reviewed journals and presented at national and international conferences.

## Discussion

### Strengths and Challenges

The VR training on safety culture and principles of HROs continue to be an appreciated topic in many other research areas, and to our knowledge, there is currently no experienced-based training to enhance HCWs’ understanding of these topics. The key strengths of this study are as follows:

1. This is a first-of-its-kind VR training to enhance HCWs’ understanding of contributing factors to patient safety events.
2. This is also a first-of-its-kind VR training to enhance HCWs’ sensemaking of patient safety culture and principles and behaviors of HROs.
3. This study uses SCM, HFACS, and sensemaking theoretical frameworks to guide the development and evaluation of VR training.
4. It involves a multidisciplinary team and promotes collaboration among clinicians, human factors engineers, and multimedia and technology innovation experts.
5. We focus on the department of surgery, which is an innovative environment owing to the complexity of the department, where multiple factors can contribute to the occurrence of a patient safety event (eg, technical and teamwork skills, communication, technology, hierarchy) as well as the high impact of patient safety events.

We recognize that implementation of the training and having most of the department of surgery staff taking the VR training will be challenging. We have already secured the leadership’s commitment to supporting this training. Based on our sample size calculations, we need 80 to agree to participate in our VR training, which is reasonable. In our past research, we were able to successfully recruit >80% of staff from various departments at UNC. Further, unanticipated factors (eg, changes in clinical practice, patient volumes, COVID-19 surges) impacting the culture of patient safety, TeamSTEPPS behaviors, and reporting of patient safety events are expected. Surgery department leadership shall inform the research team of any interventions that take place during/after the implementation of VR training, and we will account for them in our statistical analysis.

### Scalability and Sustainability

The findings and experience gained by the research team from conducting this study will apply broadly to other departments in UNC Health (eg, neurosurgery, urology, orthopedics, obstetrics-gynecology) as well as other similar surgery departments nationally. We will expand the entire concept to film additional scenarios to capture other contexts (eg, intensive care unit procedures, care transitions, imaging, laboratory studies). Ultimately, if proven successful, VR training could be integrated into training curricula for attending continuous education, resident and student education, including medical and nursing populations. We also plan to work with the UNC Health Office of Quality Excellence and UNC Training and Education to help guide the implementation of our VR training more broadly.

### Conclusions

To date, no research has been conducted into the effectiveness of VR in improving patient safety culture and HCWs’ understanding of contributing factors of patient safety events and sensemaking of patient safety culture and principles and behaviors of HROs. Our study will be the first to assess the effect of VR-based training on patient safety culture, TeamSTEPPS behavior scores, and reporting of patient safety events in the clinical environment of the department of surgery. Based on the results, VR training can be further developed to improve patient safety culture in other specialties. In addition, the foundation that will be laid with this study allows us to design follow-up studies, for example, to compare the effectiveness of VR with other modes of training.

## Appendix

Multimedia Appendix 1 Participants’ understanding of the contributing factors to an event using a scale based on the human factors analysis and classification system.

Multimedia Appendix 2 Interview questions.

Multimedia Appendix 3 Written description of the recorded patient safety event.

Multimedia Appendix 4 Peer-review report from the Center for Health Innovation.

## Abbreviations

AHRQ: Agency for Healthcare Research and Quality
HCW: health care worker
HFACS: Human Factors Analysis and Classification System
HRO: high reliability organization
SCM: Swiss cheese model
SOPS: Survey on Patient Safety Culture
TENTS: Teamwork Evaluation of Non-Technical Skills
UNC: University of North Carolina
VR: virtual reality
