# Supplementary material for: A Novel Theory-Based Virtual Reality Training to Improve Patient Safety Culture in the Department of Surgery of a Large Academic Medical Center: Protocol for a Mixed Methods Study
Source: JMIR Res Protoc. 2022 Aug 24;11(8):e40445. doi: 10.2196/40445 (PMC9453584; doi:10.2196/40445)
Supplement: Multimedia Appendix 1 [file resprot_v11i8e40445_app1.docx]

**Multimedia Appendix 1. Participants’ understanding of the contributing factors to an event using a scale based on the human factors analysis and classification system.**

Please rate the following factors on their contribution to lead up to the adverse event you just experienced.

1. **Organizational influences**
2. *Organizational climate*: Prevailing atmosphere/vision within the organization including such things as policies, command structure, and culture.
3. *Operational process:* Formal process by which the vision of an organization is carried out including operations, procedures, and oversight among others.
4. *Resource management:* How human, monetary, and equipment resources necessary to carry out the vision are managed.
5. **Unsafe supervision**
6. *Inadequate supervision:* Oversight and management of personnel and resources including training, professional guidance, and operational leadership among other aspects.
7. *Planned inappropriate operations:* Management and assignment of work including aspects of risk management, crew pairing, operational tempo, etc.
8. *Failed to correct known problems:* Those instances when deficiencies among individuals, equipment, training, or other related safety areas are ‘‘known” to the supervisor, yet are allowed to continue uncorrected.
9. *Supervisory violations:* The willful disregard for existing rules, regulations, instructions, or standard operating procedures by management during the course of their duties
10. **Preconditions for unsafe acts**

Environmental Factors

1. *Technological Environment:* This category encompasses a variety of issues including the design of equipment and controls, display/interface characteristics, checklist, layouts, task factors, and automation.
2. *Physical Environment:* Included are both the operational setting (e.g., weather, altitude, terrain) and the ambient environment, such as heat, vibration, lighting, and toxins.

Condition of the operator

1. *Adverse mental states:* Acute psychological and/or mental conditions that negatively affect performance such as mental fatigue, pernicious attitudes, and misplaced motivation
2. *Adverse physiological states:* Acute medical and/or physiological conditions that preclude safe operations such as illness, intoxication, and the myriad of pharmacological and medical abnormalities known to affect performance
3. *Physical/mental limitations*: Permanent physical/mental disabilities that may adversely impact performance such as poor vision, lack of physical strength, mental aptitude, general knowledge, and a variety of other chronic mental illnesses

Personnel factors

1. *Crew resource management*: Includes a variety of communication, coordination, and teamwork issues that impact performance
2. *Personal readiness:* Off-duty activities required to perform optimally on the job such as adhering to crew rest requirements, alcohol restrictions, and other off-duty mandates
3. **Unsafe acts**

Errors

1. *Decision errors*: These ‘‘thinking” errors represent conscious, goal-intended behavior that proceeds as designed, yet the plan proves inadequate or inappropriate for the situation. These errors typically manifest as poorly executed procedures, improper choices, or simply the misinterpretation and/or misuse of relevant information.
2. *Skill-based errors:* Highly practiced behavior that occurs with little or no conscious thought. These ‘‘doing” errors frequently appear as a breakdown in visual scan patterns, inadvertent activation/deactivation of switches, forgotten intentions, and omitted items in checklists often appear. Even the manner or technique with which one performs a task is included.
3. *Perceptual errors:* These errors arise when sensory input is degraded, as is often the case when flying at night, in poor weather, or in otherwise visually impoverished environments. Faced with acting on imperfect or incomplete information, aircrew runs the risk of misjudging distances, altitude, and decent rates, as well as responding incorrectly to a variety of visual/vestibular illusions.

Violations

1. *Routine violations*: Often referred to as ‘‘bending the rules” this type of violation tends to be habitual by nature and is often enabled by a system of supervision and management that tolerates such departures from the rules.
2. *Exceptional violations:* Isolated departures from an authority, neither typical of the individual nor condoned by management.
