# Supplementary material for: A Novel Theory-Based Virtual Reality Training to Improve Patient Safety Culture in the Department of Surgery of a Large Academic Medical Center: Protocol for a Mixed Methods Study
Source: JMIR Res Protoc. 2022 Aug 24;11(8):e40445. doi: 10.2196/40445 (PMC9453584; doi:10.2196/40445)
Supplement: Multimedia Appendix 2 [file resprot_v11i8e40445_app2.docx]

**Multimedia Appendix 2. Interview questions.**

Thank you for taking the time to speak with us today. We are interested in learning about your experience while reading the patient safety case/during the virtual reality patient safety training and your suggestions on how we can further improve it. The questions we are going to ask you don’t have right or wrong answers.

Your participation is strictly voluntary, and you are in no way required to provide information if you choose not to at any point. We are audio recording and taking notes during the interview so we can review what you have said at a later time. Our entire conversation today will be kept strictly confidential and no identifying information (i.e. your name) will be associated with your responses. The interview will take approximately 20 minutes. Do you have any questions before we begin?

1. Can you please describe what happened?
   1. What led to the patient bleeding in the OR?
   2. Who was involved?
   3. What actions occurred?
   4. What were the team members thinking and feeling?
   5. Whose fault was it?
2. What there anything you found hard to understand?
3. Was there something about the safety culture or the operating room culture that led to that particular event?
4. Are there aspects of the patient safety culture that promote doing the wrong things?
5. Is there a process that tends to engage people in risky workarounds or risky behaviors?
6. What interventions can be put in place to ensure necessary supplies are available?
   1. How will you reduce it happening again?
   2. How will you know the risk is reduced?
