# Supplementary material for: A Novel Theory-Based Virtual Reality Training to Improve Patient Safety Culture in the Department of Surgery of a Large Academic Medical Center: Protocol for a Mixed Methods Study
Source: JMIR Res Protoc. 2022 Aug 24;11(8):e40445. doi: 10.2196/40445 (PMC9453584; doi:10.2196/40445)
Supplement: Multimedia Appendix 3 [file resprot_v11i8e40445_app3.docx]

**Multimedia Appendix 3.** **Written description of the recorded patient safety event.**

Mr. Smith is a 33-year-old male with a PMH of an ATV accident in 2019 leading to an R femur fx s/p ORIF, which was complicated by RLE DVT (for which he is currently on Xarelto) and a bucket-handle mesenteric injury s/p ex-lap and repair. He was recently seen in the clinic for a symptomatic ventral hernia without evidence of incarceration and is scheduled for an open hernia repair. Vital signs pre-op are T: 37.4, HR: 94, BP: 140/74, SpO2: 100% on RA.

During the case, the hernia was successfully reduced. Moderate sanguineous output was appreciated in the JP drain while closing by the resident. The attending notes it and leaves the OR, while the PGY 2 resident finishes closing the incision.

After closing, the resident leaves the OR and asks the circulating nurse to finish dressing the incision site as she leaves. The CN asks for a debrief, and the resident states that there were no complications.

The CN notes continuous sanguineous output filling the JP drain and verbalizes his concern to the scrub tech. The scrub tech continues to count his table and does not address the bleeding. The CN verbalizes that the patient’s abdomen is rigid on palpation, and his heart rate is now tachycardic in the 130’s. The anesthesia resident states that the patient is waking up and starts to push 100 mcg’s of Fentanyl. While pushing the med, the cardiac monitor alarms and the anesthesia resident states that the patient is in V-tach. The scrub tech feels no femoral pulse on the patient. Anesthesia calls a code blue, while the scrub tech starts CPR. The surgical team is called back into the OR.

During the code, the anesthesia resident is pressure bagging crystalloid into the patient. He asks for blood products, but there is none in the OR. When they do their next pulse check, the monitor shows V-fib. They shock the patient. The monitor reads asystole. They find no pulse. They have been running the code for 45 minutes. Attending calls time of death.
