# Supplementary material for: A Novel Theory-Based Virtual Reality Training to Improve Patient Safety Culture in the Department of Surgery of a Large Academic Medical Center: Protocol for a Mixed Methods Study
Source: JMIR Res Protoc. 2022 Aug 24;11(8):e40445. doi: 10.2196/40445 (PMC9453584; doi:10.2196/40445)
Supplement: Multimedia Appendix 4 [file resprot_v11i8e40445_app4.pdf]

2022 Innovation Pilot Awards – Peer Review Results*

CENTER FOR HEALTH INNOVATION

Awardee/PI: Lukasz Mazur, PhD
Project Title: Virtual reality patient safety training to enhance the holistic view of the culture of patient safety and high-reliability organizations
Project Period: 6/1/2022 – 5/30/2023

Proposal Average Score: 2.3

Proposal Feedback Summary:

> *The reviewers appreciated the innovative nature of the project and noted the existing efforts already made around developing a script and recruiting actors. They agreed that this training approach would likely resonate with younger clinicians and could help improve outcomes related to patient safety. Some concerns were raised around a lack of detail on how the software for the program would be developed and the advantages to using VR over the existing simulation trainings. Overall, the reviewers felt investing in this technology would validate VR as another viable modality for training and increase access to simulation beyond clinicians.*

Scoring Levels:

1 Exceptional--Exceptionally strong with essentially no weaknesses
2 Outstanding--Extremely strong with negligible weaknesses
3 Excellent--Very strong with only some minor weaknesses
4 Very good--Strong but with numerous minor weaknesses
5 Good--Strong but with at least one moderate weaknesses
6 Satisfactory--Some strengths but also some moderate weaknesses
7 Fair--Some strengths but with at least one major weakness
8 Marginal--A few strengths and a few major weaknesses
9 Poor--Very few strengths and numerous major weaknesses

* The Innovation Pilot Award Review panel consists of faculty and administrators from the UNC School of Medicine and executives from UNC Health.

UNC HEALTH

UNC SCHOOL OF MEDICINE
